# ceRNA network-regulated COL1A2 high expression correlates with poor prognosis and immune infiltration in colon adenocarcinoma

**DOI:** 10.1038/s41598-023-43507-x

**Published:** 2023-10-07

**Authors:** Xia Yuan, Yi He, Wei Wang

**Affiliations:** 1 Gastroenterology and Urology Department II, Hunan Cancer Hospital/The Affiliated Cancer Hospital of Xiangya School of Medicine, Central South University, No. 283, Tongzipo Road, Changsha, 410013 People’s Republic of China; 2 Clinical Research Center for Gastrointestinal Cancer in Hunan Province, Changsha, People’s Republic of China

**Keywords:** Cancer, Gastrointestinal cancer, Colorectal cancer

## Abstract

Collagen type I α 2 (COL1A2) is a major component of collagen type I. Recently, abnormal COL1A2 expression has been reported in human cancers. However, the specific role and mechanism of COL1A2 in colon adenocarcinoma (COAD) remain unclear. We performed the pan-cancer analysis of COL1A2 expression in 33 types of human cancers from TIMER database and integrated data combined TCGA with GTEx. The prognostic values of COL1A2 for 17 cancer types of interest were estimated from GEPIA database. The results showed that COL1A2 was significantly upregulated in COAD tissues and that higher COL1A2 expression predicted unfavorable prognosis for patients with COAD. Next, COL1A2-related functional pathways in COAD were analyzed with TCGA data using R package. Additionally, we constructed a ceRNA network that LINC00638/hsa-miR-552-3p axis served as a potential regulatory pathway of COL1A2 in COAD. Furthermore, our findings showed that COL1A2 positively associated with immune infiltration and that tumor immune escape might be involved in COL1A2-mediated carcinogenesis in COAD. For the first time, we constructed a ceRNA prediction network of COL1A2 and explored the association of COL1A2 with tumor immune microenvironment remodeling. The findings may advance our understanding of the pathogenesis mechanism in COAD and paves the way for further cancer therapeutics.

## Introduction

Colon adenocarcinoma (COAD) is one of the most prevalent cancers worldwide, and is a leading cause of cancer-related deaths. Global Cancer Statistics 2020 reported that the incidence rate of colorectal cancer has exceeded that of gastric cancer, ranking first among the digestive system malignancies^1^. With lifestyles becoming increasingly sedentary and dietary patterns changing, the incidence and mortality rate of COAD are still rising and continuously increase among younger people^2^. Surgical resection is currently the only means of radical treatment, but most patients with COAD are diagnosed at an advanced stage^3^. COAD exhibits a high rate of recurrence and distant metastasis, and the lack of tumor specificity and resistance to chemotherapy remain the significant barrier for prognosis improvement^4^. The treatment options are limited for patients with advanced COAD, resulting in a poor 5-year survival rate at only 11.7%^5^. Therefore, it is warranted to explore potential therapeutic targets and identify promising prognostic biomarkers in COAD.

Type I collagen, an important member of the collagen family, is the most abundant protein of bone, skin, and tendon extracellular matrices. Type I collagen comprises two α1 chains (COL1A1) and one α2 chain (COL1A2)^6^. Previous literature primarily focused on the role of COL1A2 in osteogenesis, osteoporosis and bone diseases. It was reported that mutations in COL1A1 and COL1A2 gene caused osteogenesis imperfect, known as an autosomal dominant disorder^7^. The COL1A2 gene is widely used in experimental models for studying the molecular basis of collagen I biosynthesis^8^. Recently, type I collagen is believed to be involved in carcinogenesis and abnormal COL1A2 expression has been reported in human cancers, including gastric cancer^9,10^, and pancreatic cancer^11^. As reported, the expression of COL1A1 was significantly upregulated in colorectal cancer tissues and cell lines, associated with metastasis, serosal invasion, lymph metastases and hematogenous metastases, indicating that COL1A1 may serve as an oncoprotein in colorectal cancer^12,13^. However, Yu et al. reported that COL1A2 was significantly downregulated in primary colorectal cancer tissues and overexpressed COL1A2 inhibited proliferation, migration, and invasion of colorectal cell lines^14^. Another study identified COL1A2 as a core gene for colorectal cancer and it played important roles in tumor progression and prognosis of stage IIA colon cancer^15^. Taken together, the role of COL1A2 in colon cancer remains controversial and a comprehensive study regarding the expression, prognosis, and mechanism of COL1A2 in COAD is still absent. Moreover, the association of COL1A2 with tumor immune infiltration and immune signatures in COAD is still not determined.

**Figure 1:**
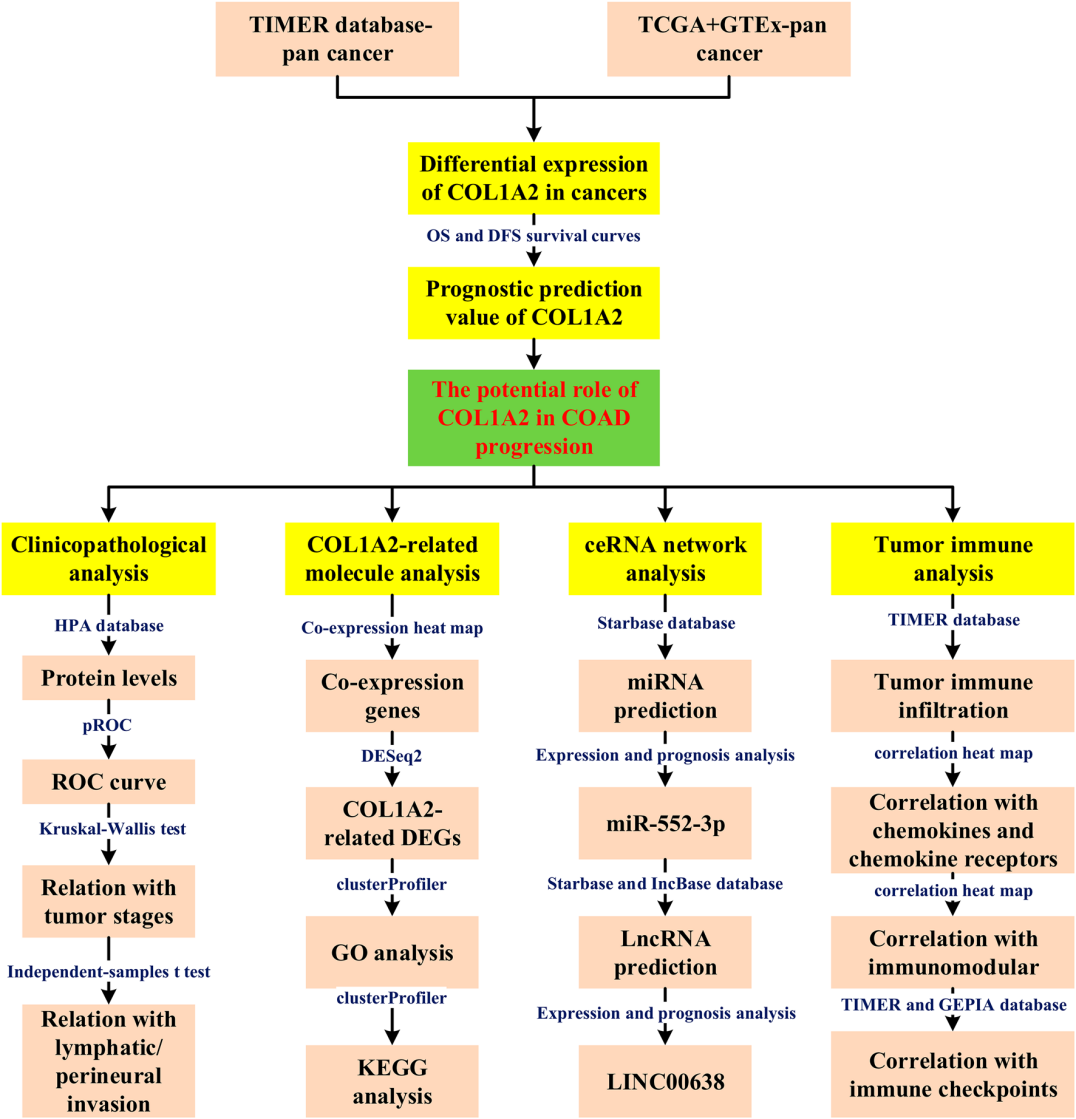
Research flow chart.

In the present study, we performed expression analysis and survival analysis for COL1A2 across 33 types of human cancer, identifying its overexpression and prognostic value in COAD. Next, comprehensive analyses were performed to explore COL1A2-related mechanisms in COAD using bioinformatic tools. We successfully constructed a ceRNA network as the upstream regulatory mechanism of COL1A2. Moreover, this research investigated the association of COL1A2 with tumor immune infiltration and immune signatures in COAD. The results suggested that tumor immune infiltration and tumor immune escape might be involved in COL1A2-medidated cancer development, providing clues for improving the immunotherapy efficacy of COAD by targeting COL1A2. To the best of our knowledge, for the first time, we predicted the ceRNA network of COL1A2 and explored its association with tumor immune microenvironment remodeling. The present study may advance our understanding of the pathogenesis of COAD and paves the way for further cancer therapeutics. The investigation process was presented as a flow chart in Fig. 1.

## Results

### Pan-cancer analysis of COL1A2 expression

**Figure 2:**
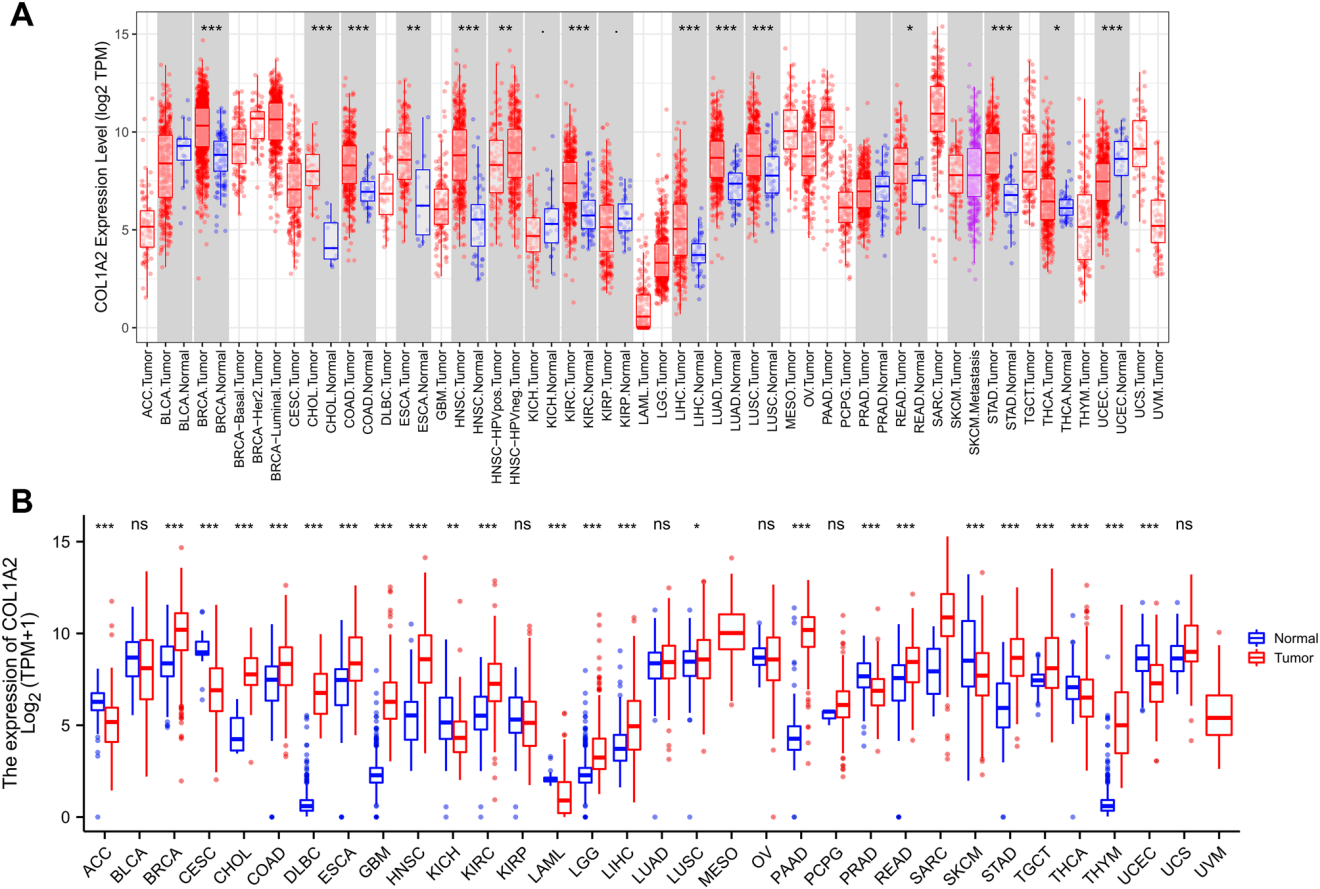
Pan-cancer analysis of COL1A2 differential expression between cancerous tissues and corresponding normal tissues. (**A**) The expression of COL1A2 in 33 types of human cancer based on TIMER database. (**B**) The expression of COL1A2 in 33 types of human cancer based on TCGA and GTEx database. *ns* not significant; *p value < 0.05; **p value < 0.01; ***p value < 0.001.

Transcriptome and multi-omics analysis are applied to the study of the pathogenesis and biological markers of various diseases, especially in tumor research^16–19^. To explore the potential role of COL1A2 in carcinogenesis, we first checked its expression based on TIMER (Tumor Immune Estimation Resource) database across 33 cancer types (i.e. ACC, BLCA, BRCA, CESC, CHOL, COAD, DLBC, ESCA, GBM, HNSC, KICH, KIRC, KIRP, LAML, LGG, LIHC, LUAD, LUSC, MESO, OV, PAAD, PCPG, PRAD, READ, SARC, SKCM, STAD, TGCT, THCA, THYM, UCEC, UCS, and UVM). As shown in Fig. 2A, compared with normal tissues, COL1A2 was significantly upregulated in 12 cancer types (i.e. BRCA, CHOL, COAD, ESCA, HNSC, KIRC, LIHC, LUAD, LUSC, READ, STAD, and THCA), but downregulated in 3 cancer types (i.e. KICH, KIRP, and UCEC). Considering the limited samples of normal tissues in TIMER, the differential expression of COL1A2 between cancers and normal tissues was further validated in the integrated databases combined The Cancer Genome Atlas (TCGA) with The Genotype-Tissue Expression (GTEx). As presented in Fig. 2B, compared with corresponding normal controls, COL1A2 was significantly upregulated in 17 cancer types (i.e. BRCA, CHOL, COAD, DLBC, ESCA, GBM, HNSC, KIRC, LGG, LIHC, LUSC, PAAD, READ, SARC, STAD, TGCT, and THYM), 7 of which had no corresponding normal controls in TIMER database (i.e. DLBC, GBM, LGG, PAAD, SARC, TGCT, and THYM). It was observed that COL1A2 was remarkably downregulated in 8 cancer types (i.e. ACC, CESC, KICH, LAML, PRAD, SKCM, THCA, and UCEC). However, 6 types of cancer tissues (i.e. BLCA, KIRP, LUAD, OV, PCPG, and UCS) shared the same level of COL1A2 expression with corresponding normal controls. Taken together, COL1A2 was upregulated in BRCA, CHOL, COAD, DLBC, ESCA, GBM, HNSC, KIRC, LGG, LIHC, LUSC, PAAD, READ, SARC, STAD, TGCT, and THYM, indicating its potential role as a crucial regulator in carcinogenesis for the 17 cancer types.

### Prognostic value of COL1A2 expression in human cancers

**Figure 3:**
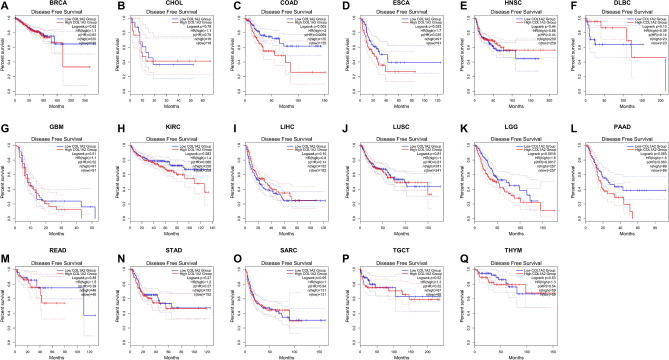
The disease-free survival (DFS) analysis for COL1A2 in various human cancers based on GEPIA database. (**A–Q**) The DFS plot for COL1A2 in BRCA (**A**), CHOL (**B**), COAD (**C**), ESCA (**D**), HNSC (**E**), DLBC (**F**), GBM (**G**), KIRC (**H**), LIHC (**I**), LUSC (**J**), LGG (**K**), PAAD (**L**), READ (**M**), STAD (**N**), SARC (**O**), TGCT (**P**), and THYM (**Q**).

**Figure 4:**
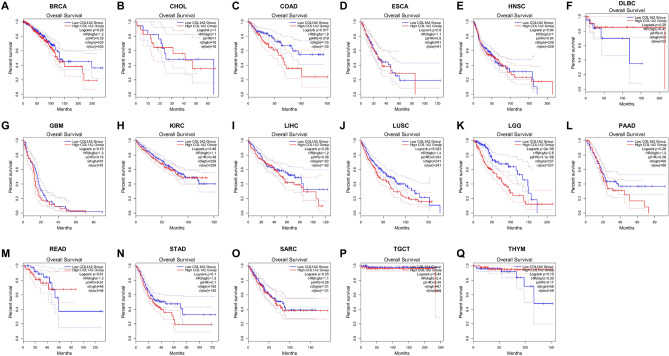
The overall survival (OS) analysis for COL1A2 in various human cancers based on GEPIA database. (**A–Q**) The OS plot for COL1A2 in BRCA (**A**), CHOL (**B**), COAD (**C**), ESCA (**D**), HNSC (**E**), DLBC (**F**), GBM (**G**), KIRC (**H**), LIHC (**I**), LUSC (**J**), LGG (**K**), PAAD (**L**), READ (**M**), STAD (**N**), SARC (**O**), TGCT (**P**), and THYM (**Q**).

Next, the prognostic value of COL1A2 expression for the 17 types of cancers were estimated, covering two prognostic indicators consisting of overall survival (OS) and disease-free survival (DFS). In terms of DFS (Fig. 3), patients with higher expression of COL1A2 had poorer prognosis for COAD and ESCA. No significant prognostic prediction value was observed in the other types of cancers. With regards to OS (Fig. 4), COL1A2 expression level was valuable for predicating prognosis of patients only in COAD and higher COL1A2 expression indicated unfavorable prognosis in COAD. There was no statistical significance for COL1A2 to predict prognosis of patients in other cancer types. Suggested by the results of OS and DFS analysis, COL1A2 may serve as an unfavorable prognostic biomarker in patients with COAD.

### Association of COL1A2 expression with clinicopathological features for COAD

**Figure 5:**
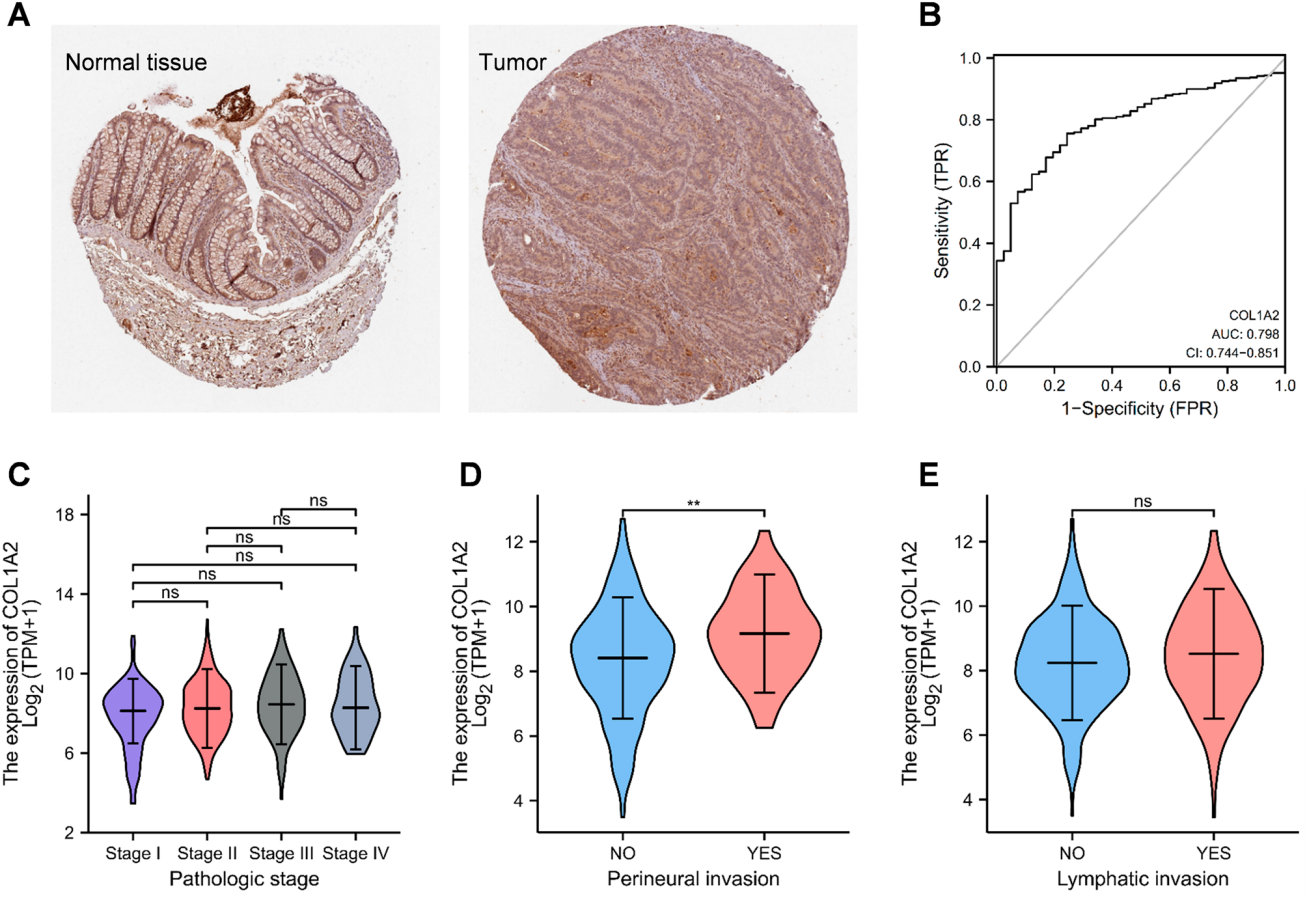
Association of COL1A2 expression with clinicopathological features in COAD. (**A**) COL1A2 expression in COAD at the protein level (IHC) based on HPA database. (**B**) A ROC curve was constructed to test the value of COL1A2 to identify COAD tissues. (**C**) The expression levels of COL1A2 in COAD tissues with different pathologic stages. (**D**) The expression levels of COL1A2 in COAD tissues with and without perineural invasion. (**E**) The expression levels of COL1A2 in COAD tissues with and without lymphatic invasion. *ns* not significant; **p value < 0.01; ***p value < 0.001.

The upregulated expression of COL1A2 in COAD was validated at the protein level by immunohistochemical (IHC) analysis based on Human Protein Atlas (HPA) database (Fig. 5A). Receiver operating characteristic (ROC) curve was constructed to estimate the distinguishing efficacy of COL1A2 expression between COAD and normal colon mucosa tissue (Fig. 5B). The area under the curve (AUC) of COL1A2 is 0.798, suggesting a moderate to strong distinguishing efficacy of COL1A2 for COAD. COL1A2 may serve as a potential valuable identification biomarker for COAD tissues. Furthermore, we investigated the correlation between COL1A2 and clinicopathological features of COAD. As shown in Fig. 5C, the expression levels of COL1A2 across different pathologic stages (I vs. II vs. III vs. IV) were not significantly different. However, overexpressed COL1A2 was significantly correlated with perineural invasion (Yes vs. No, p < 0.01) (Fig. 5D), but COL1A2 expression has no relation with lymphatic invasion (Fig. 5E). These results suggested that COADs with higher COL1A2 expression were prone to be more aggressive compared to those with low COL1A2 expression.

### Analysis of COL1A2-related molecules and functional pathways in COAD

**Figure 6:**
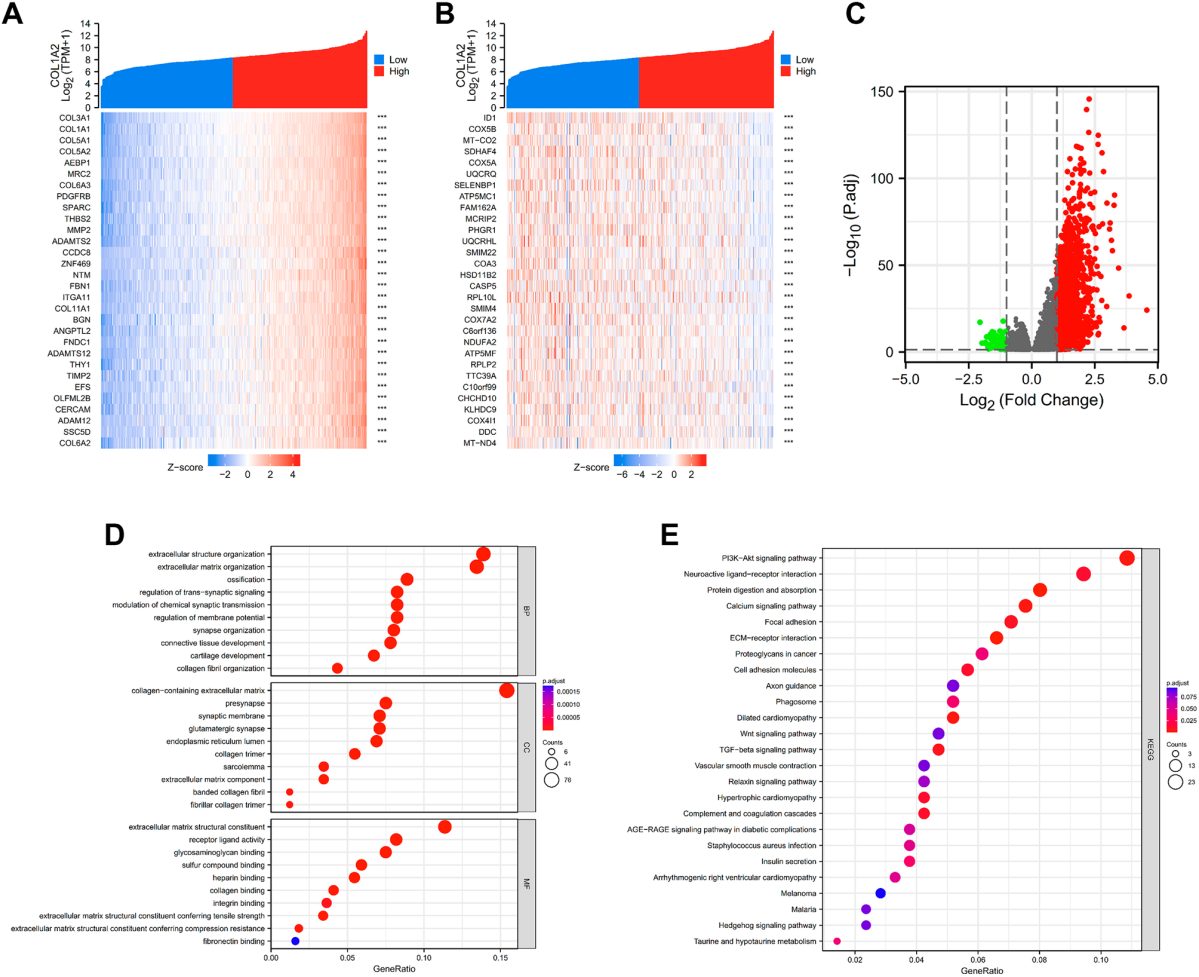
Analysis of COL1A2-related molecules and functional pathways in COAD. (**A**) The top 30 genes most positively correlated with COL1A2 in COAD were displayed in the heatmap. (**B**) The top 30 genes most negatively correlated with COL1A2 in COAD were displayed in the heatmap. (**C**) COL1A2-grouped DEGs in COAD were presented in a volcano plot. (**D**) GO analysis of the upregulated DEGs. (**E**) KEGG pathway prediction of the upregulated DEGs. R package V3.6.3 (https://www.r-project.org/) was utilized. ***p value < 0.001.

**Figure 7:**
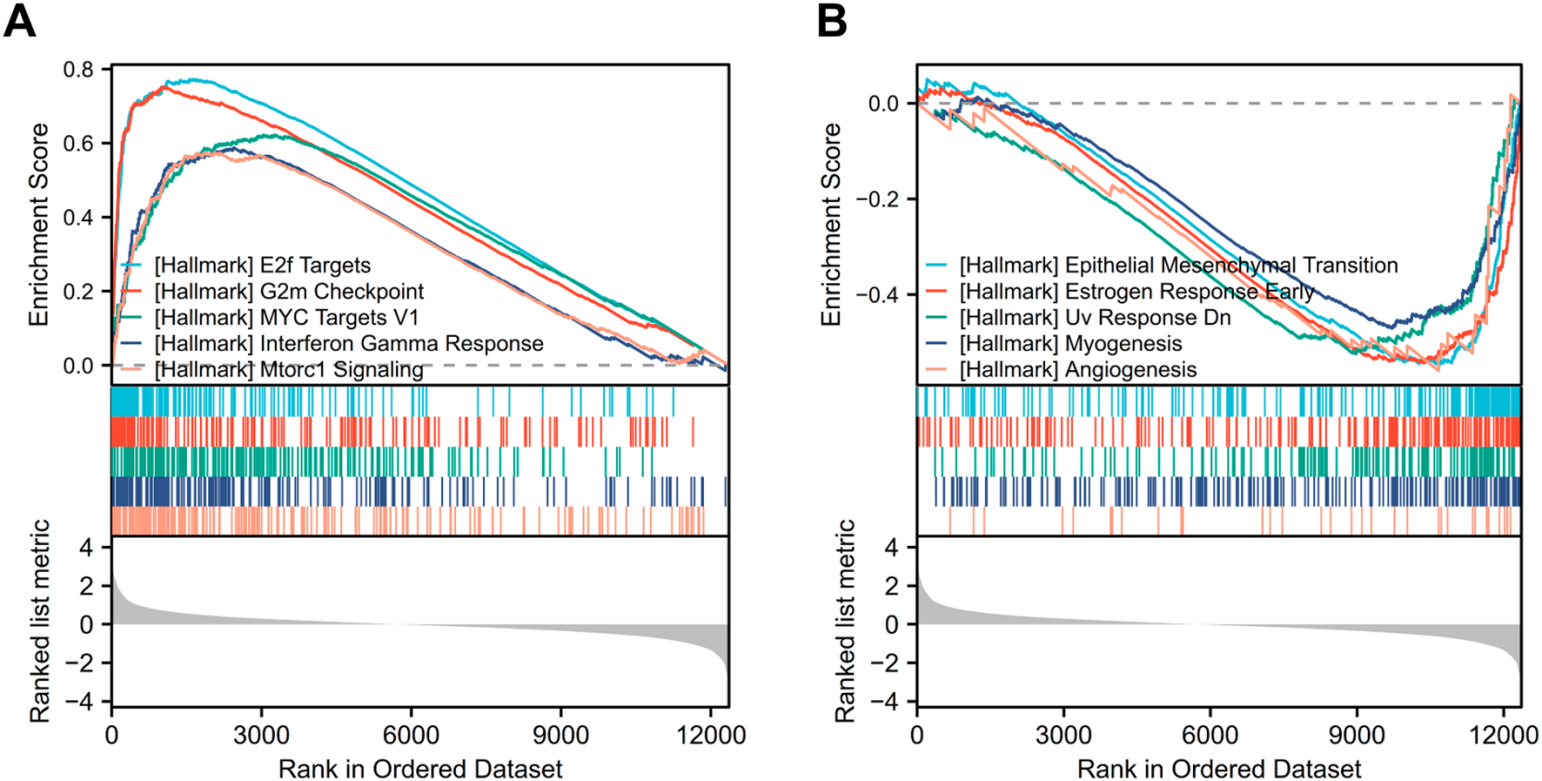
Enrichment analyses using GSEA. (**A**) Five hallmark items significantly enriched in COL1A2 high expression phenotype of COAD. (**B**) Five hallmark items significantly enriched in COL1A2 low expression phenotype of COAD (FDR < 0.25, adjusted p < 0.05).

To better understand its role and mechanism of COL1A2 in COAD tissues, the related molecules and functional pathways were investigated. First, we performed a correlation analysis between COL1A2 and other genes in COAD tissues using TCGA data. Based on the Spearman correlation coefficient, the top 30 genes most positively and negatively correlated with COL1A2 in colon cancer were identified with resulting figures displayed in the separate heatmaps (Fig. 6A,B). Next, COL1A2-grouped differentially expressed genes (DEGs) in COAD were identified with the adjusted P value < 0.05 and |log fold change [FC] > 1.5 as the threshold, and the results were presented in a volcano plot (Fig. 6C). In total, 522 upregulated and 22 downregulated DEGs were sorted out from COL1A2^high^ and COL1A2^low^ expression groups. To further explore the biological function of the upregulated DEGs, Gene Ontology (GO), Kyoto Encyclopedia of Genes and Genomes (KEGG) and Gene set enrichment analysis (GSEA) were conducted. The results of GO functional analysis and KEGG enrichment analysis have been shown below. In the biological process (BP) group, the DEGs were primarily enriched in extracellular structure organization, extracellular matrix organization, and ossification (Fig. 6D). In the cellular component (CC) group, the DEGs were primarily enriched in collagen-containing extracellular matrix, presynapse, and synaptic membrane (Fig. 6D). In the molecular function (MF) group, the genes were primarily enriched in extracellular matrix structural constituent, receptor ligand activity, and glycosaminoglycan binding (Fig. 6D). The results of KEGG pathway analysis showed that the DEGs were significantly enriched in the PI3K-Akt signaling pathway, neuroactive ligand-receptor interaction, and protein digestion and absorption (Fig. 6E). Five hallmark items, including E2F_TARGETS, G2M_CHECKPOINT, MYC_TARGETS_V1, INTERFERON_GAMMA_RESPONSE and MTORC1_SIGNALING, showed significantly differential enrichment in COL1A2 high expression phenotype (Fig. 7A); Five hallmark items, including EPITHELIAL_MESENCHYMAL_TRANSITION, STROGEN_RESPONSE_EARLY, UV_RESPONSE_DN, MYOGENESIS and ANGIOGENESIS showed significantly differential enrichment in COL1A2 low expression phenotype (Fig. 7B).

### Construction of a network of mRNA-miRNA-lncRNA

**Figure 8:**
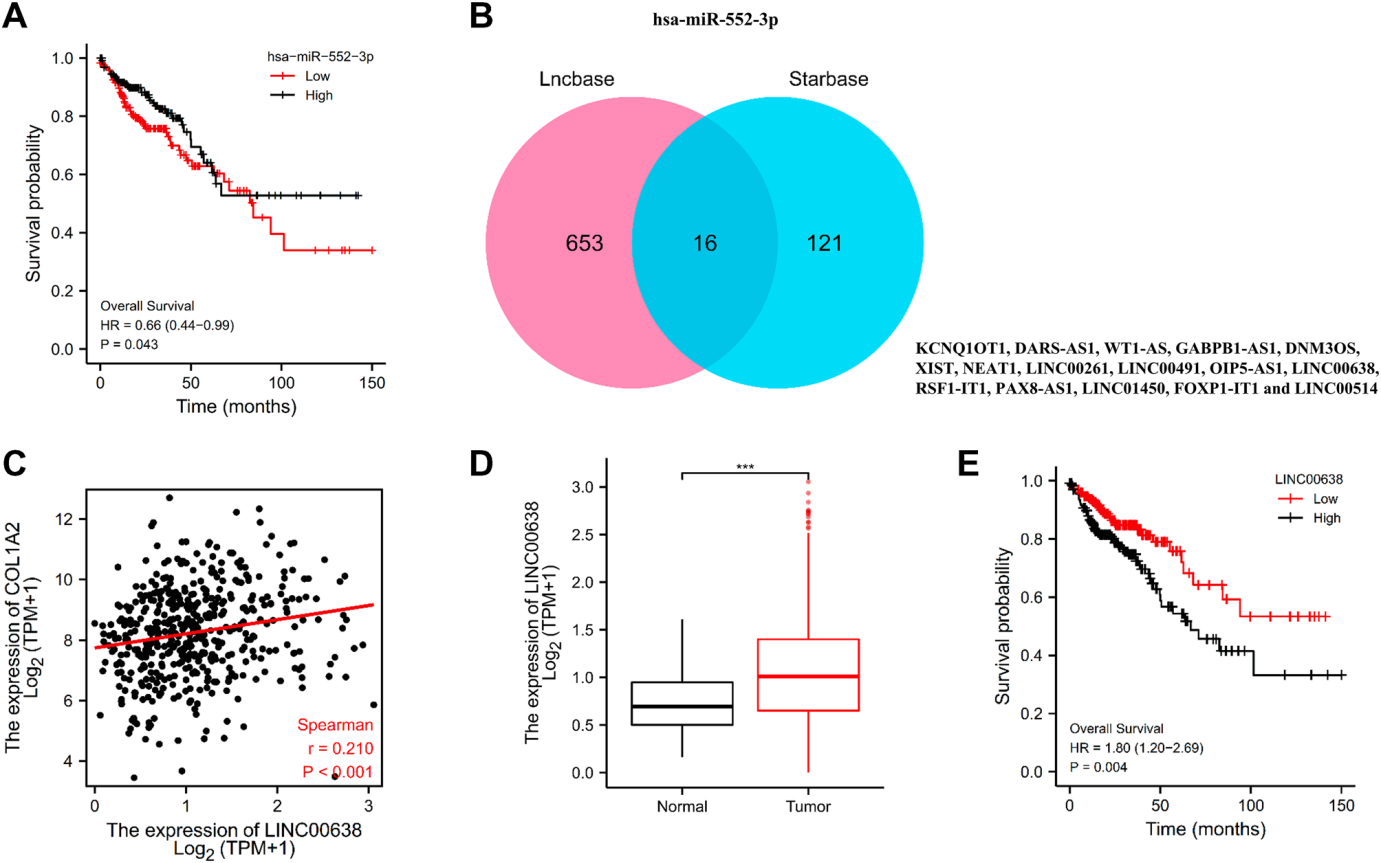
Construction of a Network of mRNA-miRNA-lncRNA. (**A**) The prognostic value of hsa-miR-552-3p expression for overall survival of COAD patients. (**B**) The intersection of miRNA reverse predicted lncRNAs based on Starbase and LncBase databases. (**C**) Correlation analysis of LINC00638 with COL1A2 expression in COAD. (**D**) The expression levels of LINC00638 in tumor and normal samples for COAD. (**E**) The prognostic value of LINC00638 expression for overall survival of COAD patients. ***p value < 0.001.

It is widely acknowledged that non-coding RNAs (ncRNAs) are responsible for the regulation of gene expression. Guided by the ceRNA hypothesis, we first predicted upstream miRNAs that could potentially bind to COL1A2 based on StarBase database and a total of 43 miRNAs targeting mRNA were checked (Table 1). Among them, a number of 24 miRNAs were negatively correlated with COL1A2 expression in COAD. Out of the 24 listed miRNAs, only one miRNA, has-miR-552-3p, was valuable for predicating prognosis of patients with COAD. Higher expression of has-miR-552-3p indicated favorable OS for patients with COAD (Fig. 8A). Guided by the miRNA-lncRNA interactions, we employed hsa-miR-552-3p to conduct reverse prediction of its upstream lncRNAs, and constructed a miRNA-lncRNA network based on the Starbase and LncBase Predicted v.2 databases. The intersection of the two databases was used to identify a total of 16 candidate lncRNAs (KCNQ1OT1, DARS-AS1, WT1-AS, GABPB1-AS1, DNM3OS, XIST, NEAT1, LINC00261, LINC00491, OIP5-AS1, LINC00638, RSF1-IT1, PAX8-AS1, LINC01450, FOXP1-IT1 and LINC00514) (Fig. 8B). Among the identified genes, only LINC00638 significantly reduced the OS of COAD patients (Fig. 8E). LINC00638 exhibited a significant positive correlation with COL1A2 expression in COAD (Fig. 8C), and the expression levels of LINC00638 in tumor and normal samples was remarkably different (Fig. 8D).

**Table 1:** Correlation analysis between COL1A2 and microRNA in COAD determined by Starbase database.

Gene name	miRNA name	R value	p value
COL1A2	hsa-let-7a-5p	0.14^a^	2.96E−03
COL1A2	hsa-let-7b-5p	0.176^a^	1.75E−04
COL1A2	**hsa-let-7d-5p**	− **0.277**^a^	**2.23E−09**
COL1A2	hsa-let-7e-5p	0.402^a^	6.43E−19
COL1A2	hsa-let-7f-5p	0.085	7.26E−02
COL1A2	**hsa-miR-19a-3p**	− **0.337**^a^	**1.94E−13**
COL1A2	**hsa-miR-19b-3p**	− **0.38**^a^	**6.29E−17**
COL1A2	**hsa-miR-25-3p**	− **0.195**^a^	**3.25E−05**
COL1A2	hsa-miR-26a-5p	− 0.012	**8.07E−01**
COL1A2	**hsa-miR-26b-5p**	− **0.287**^a^	**5.23E−10**
COL1A2	**hsa-miR-29a-3p**	− **0.258**^a^	**2.80E−08**
COL1A2	**hsa-miR-32-5p**	− **0.35**^a^	**2.09E−14**
COL1A2	**hsa-miR-92a-3p**	− **0.26**^a^	**2.23E−08**
COL1A2	**hsa-miR-98-5p**	− **0.107**^a^	**2.27E−02**
COL1A2	**hsa-miR-29b-3p**	− **0.404**^a^	**3.88E−19**
COL1A2	**hsa-miR-105-5p**	− **0.1**^a^	**3.39E−02**
COL1A2	**hsa-miR-196a-5p**	− **0.34**^a^	**8.15E−14**
COL1A2	**hsa-miR-7-5p**	− **0.158**^a^	**7.42E−04**
COL1A2	**hsa-miR-183-5p**	− **0.275**^a^	**3.17E−09**
COL1A2	**hsa-let-7 g-5p**	− **0.338**^a^	**1.65E−13**
COL1A2	hsa-miR-23b-3p	− 0.024	6.11E−01
COL1A2	hsa-miR-153-3p	− 0.067	1.55E−01
COL1A2	**hsa-miR-186-5p**	− **0.37**^a^	**4.93E−16**
COL1A2	**hsa-miR-193a-3p**	− **0.111**^a^	**1.86E−02**
COL1A2	hsa-miR-29c-3p	0.044	3.50E−01
COL1A2	hsa-miR-363-3p	0.071	1.31E−01
COL1A2	hsa-miR-342-3p	0.037	4.31E−01
COL1A2	**hsa-miR-196b-5p**	− **0.233**^a^	**5.74E−07**
COL1A2	hsa-miR-193b-3p	0.069	1.45E−01
COL1A2	hsa-miR-524-5p	0.007	8.89E−01
COL1A2	hsa-miR-520d-5p	0.005	9.18E−01
COL1A2	**hsa-miR-552-3p**	− **0.188**^a^	**5.78E−05**
COL1A2	hsa-miR-92b-3p	0.008	8.71E−01
COL1A2	hsa-miR-579-3p	− 0.057	2.24E−01
COL1A2	**hsa-miR-584-5p**	− **0.162**^a^	**5.43E−04**
COL1A2	**hsa-miR-625-5p**	− **0.235**^a^	**4.48E−07**
COL1A2	**hsa-miR-642a-5p**	− **0.129**^a^	**6.07E−03**
COL1A2	hsa-miR-371a-5p	− 0.043	3.65E−01
COL1A2	**hsa-miR-513b-5p**	− **0.193**^a^	**3.75E−05**
COL1A2	hsa-miR-1323	− 0.004	9.37E−01
COL1A2	hsa-miR-548o-3p	− 0.021	6.52E−01
COL1A2	**hsa-miR-4766-5p**	− **0.094**^a^	**4.68E−02**
COL1A2	hsa-miR-873-3p	0.02	6.78E−01

A p value of less than 0.05 defined statistical significance.

^a^These results are statistically significant.

Bold fonts indicate the significantly negative correlation between COL1A2 and miRNAs in COAD.

### Correlation analysis of COL1A2 with tumor immune infiltration in COAD

**Figure 9:**
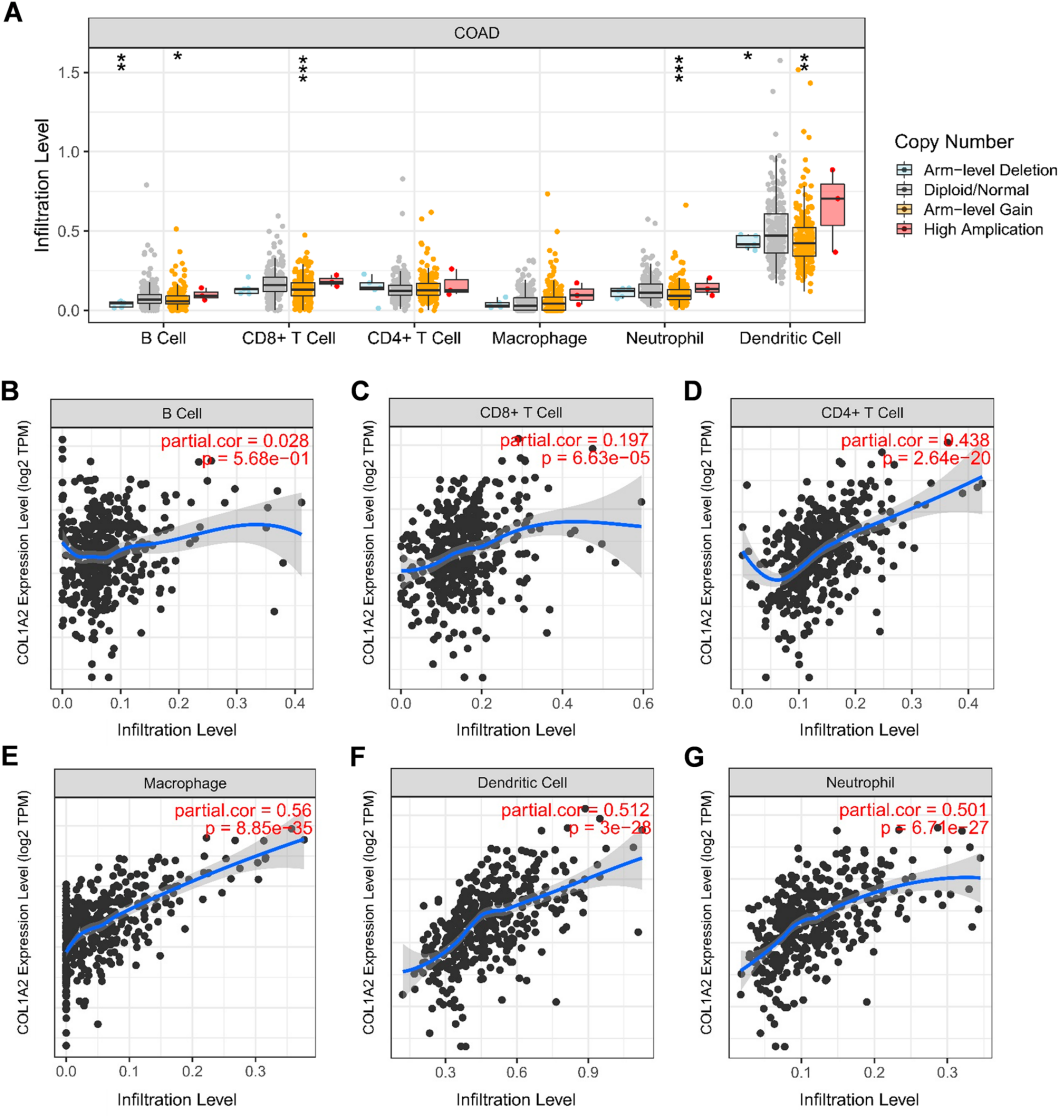
Correlation analysis of COL1A2 with tumor immune infiltration in COAD. (**A**) The levels of immune cell infiltration under various copy numbers of COL1A2 in COAD. (**B–G**) Correlation of COL1A2 expression with the abundance of B cell (**B**), CD8+ T cells (**C**), CD4+ T cells (**D**), macrophages (**E**), dendritic cells (**F**) and neutrophils (**G**) in COAD. *p value < 0.05; **p value < 0.01; ***p value < 0.001.

Immune cells are one of the major cellular components in the tumor microenvironment, critically impacting tumor progression and response to therapy. To the best of our knowledge, there have been no previous studies that reported the role of COL1A2 in tumor immune infiltration. As shown in Fig. 9A, the levels of immune cell infiltration varied according to the copy numbers of COL1A2 in COAD, including B cells, CD8+ T cells, neutrophils, and dendritic cells. This indicates the potential role of COL1A2 expression in regulating tumor immune abundance. Next, we further investigated the correlation between COL1A2 expression and tumor immune cell infiltrates against tumor purity based on TIMER database. No significant correlation was observed between B cell infiltration level and COL1A2 expression in COAD (Fig. 9B), while increased COL1A2 expression was positively associated with the abundance of CD8+ T cells (Fig. 9C), CD4+ T cells (Fig. 9D), macrophages (Fig. 9E), dendritic cells (Fig. 9F) and neutrophils (Fig. 9G) in COAD. Moreover, we investigated the correlation between COL1A2 expression and immune cell biomarkers in COAD using the TIMER database. As shown in Table 2, COL1A2 was significantly and positively correlated with biomarkers of B cell (CD79A), CD8+ T (CD8A), CD4+ T (CD4), M1 macrophage (IRF5 and PTGS2), M2 macrophage (CD163, VSIG4, and MS4A4A), neutrophil (ITGAM and CCR7) and dendritic cell (HLA-DPB1, HLA-DQB1, HLA-DRA, HLA-DPA1, CD1C, NRP1, and ITGAX). Meanwhile, COL1A2 was significantly and negatively correlated with two biomarkers, NOS2 and CEACAM8, which were biomarkers of M1 macrophage and neutrophil, respectively. These findings partially supported that COL1A2 positively associated with immune cell infiltration in COAD.

**Table 2:** Correlation analysis between COL1A2 and biomarkers of immune cells in colon cancer determined by TIMER database.

Immune cell	Biomarker	R value	p value
B cell	CD19	0.078	1.18E−01
	CD79A	0.175^a^	3.86E−04
CD8+ T cell	CD8A	0.179^a^	2.96E−04
	CD8B	0.094	5.79E−02
CD4+ T cell	CD4	0.492^a^	4.23E−26
M1 macrophage	NOS2	− 0.159^a^	1.29E−03
	IRF5	0.301^a^	5.71E−10
	PTGS2	0.196^a^	6.90E−05
M2 macrophage	CD163	0.62^a^	1.53E−44
	VSIG4	0.549^a^	2.56E−33
	MS4A4A	0.527^a^	2.22E−30
Neutrophil	CEACAM8	− 0.182^a^	2.27E−04
	ITGAM	0.651^a^	2.74E−50
	CCR7	0.23^a^	2.71E−06
Dendritic cell	HLA-DPB1	0.424^a^	3.50E−19
	HLA-DQB1	0.212^a^	1.64E−05
	HLA-DRA	0.353^a^	2.36E−13
	HLA-DPA1	0.392^a^	2.37E−16
	CD1C	0.239^a^	1.16E−06
	NRP1	0.731^a^	4.88E−69
	ITGAX	0.644^a^	5.42E−49

A p value of less than 0.05 defined statistical significance.

^a^These results are statistically significant.

### Correlation analysis of COL1A2 with immune signatures in COAD

**Figure 10:**
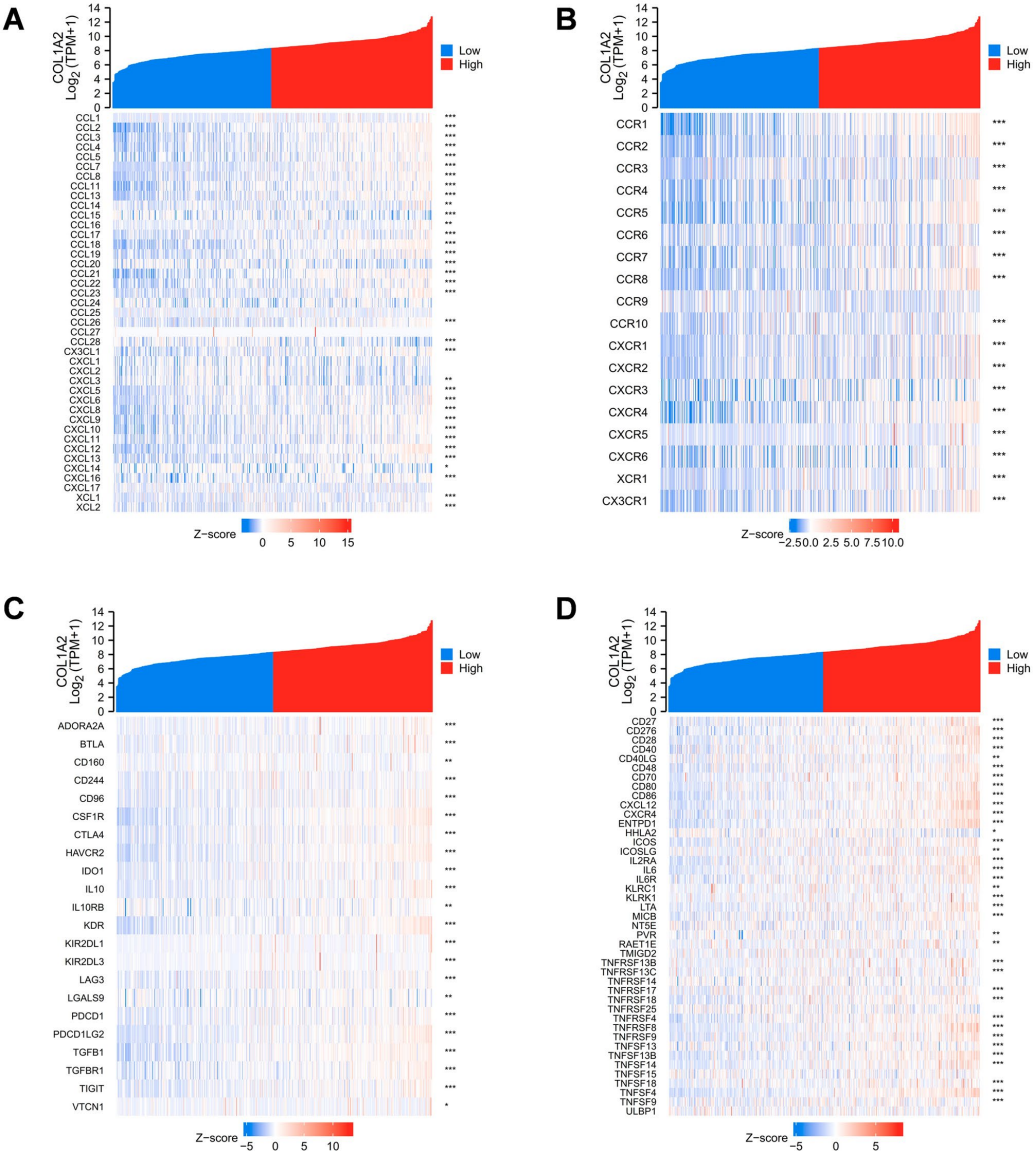
Correlation analysis of COL1A2 with various immune signatures in COAD. (**A**) Chemokines. (**B**) Chemokine receptors. (**C**) Immunoinhibitors. (**D**) Immunostimulators. *p value < 0.05; **p value < 0.01; ***p value < 0.001.

**Figure 11:**
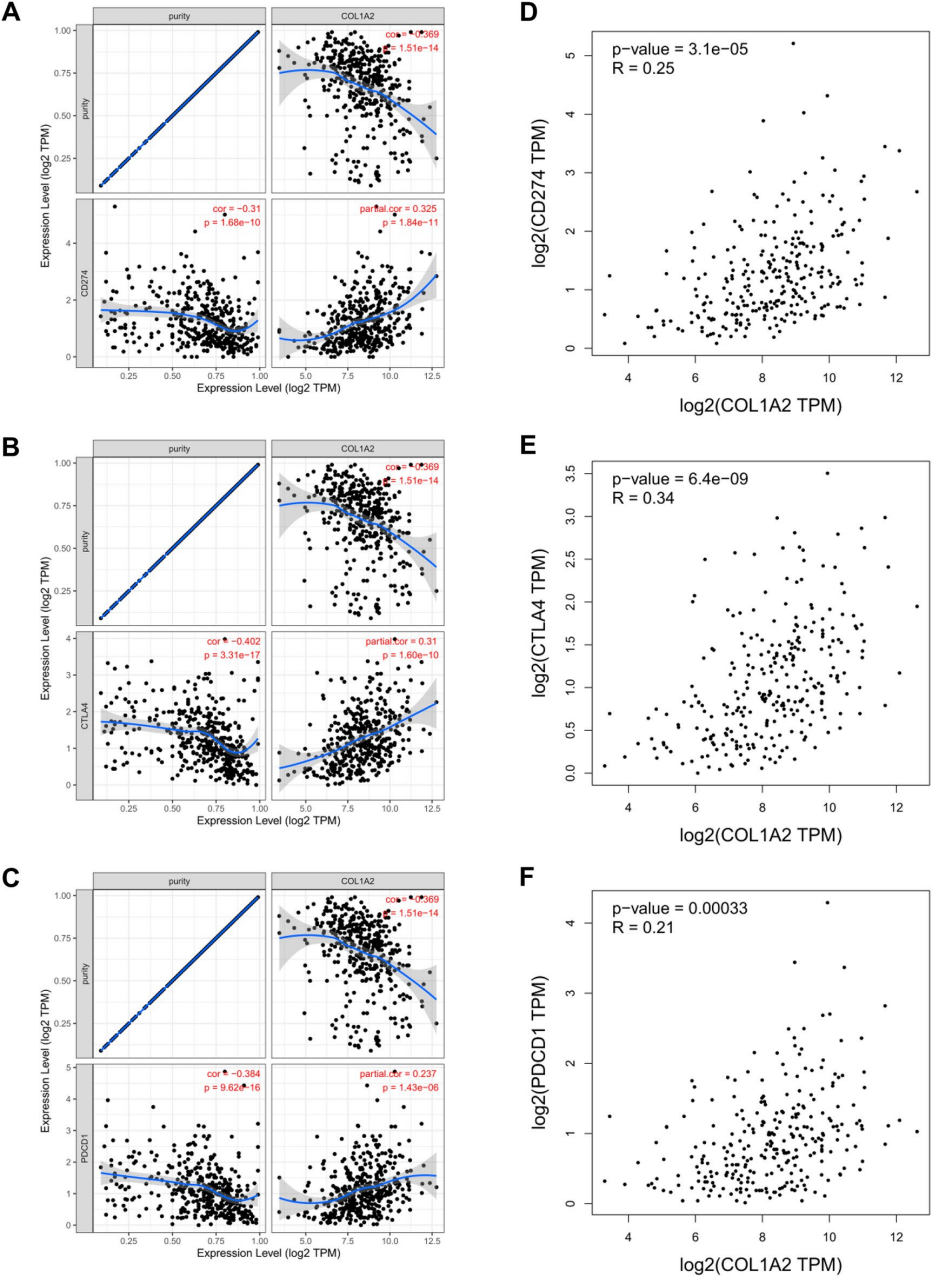
Correlation analysis of COL1A2 with important immune checkpoints in COAD. (**A**) CD274 based on TIMER database. (**B**) CTLA-4 based on TIMER database. (**C**) PDCD1 based on TIMER database. (**D**) CD274 based on GEPIA database. (**E**) CTLA-4 based on GEPIA database. (**F**) PDCD1 based on GEPIA database.

Finally, to gain a deeper understanding of the correlation between COL1A2 and immune infiltration, we performed correlation expression analyses between COL1A2 and various immune signatures, including chemokines, chemokine receptors, and two kinds of immunomodulators consisting of immunoinhibitors and immunostimulators. Heatmaps were generated to illustrate the associations between COL1A2 expression and immune signatures in COAD. The results showed that generally COL1A2 was positively associated with chemokines (Fig. 10A) and chemokine receptors (Fig. 10B). Interestingly, as Fig. 10C,D shown, COL1A2 positively correlated with common immunoinhibitor and immunostimulator molecules, suggesting the dual role of COL1A2 in tumor immune microenvironment of COAD. PD1/PD-L1 and CTLA-4 are critical immune checkpoints that play a vital role in tumor immune escape. Emphatically, we performed correlation expression analysis based on TIMER and GEPIA (Gene Expression Profiling Interactive Analysis) database to validate the associations between COL1A2 and immune checkpoints (CD274, CTLA-4, and PDCD1). As shown in Fig. 11A–C, COL1A2 was positively correlated with CD274, CTLA-4, and PDCD1 based on TIMER data which was adjusted by tumor purity. Similar results were obtained based on GEPIA database that there were positive associations between COL1A2 and CD274, CTLA-4, and PDCD1 (Fig. 11D–F). These findings suggested that tumor immune escape might be involved in COL1A2-mediated carcinogenesis of COAD.

## Discussion

COAD is one of the most commonly diagnosed malignancies and the leading cause of cancer-related deaths in the world, with increasing incidence and mortality^1^. COAD exhibits a high rate of recurrence, distant metastasis, and resistance to conventional therapy. The efficacy of immune checkpoint inhibitor therapy on COAD remains limited. Effective targeted drugs and prognostic markers are lacking for COAD, and it is warranted to develop effective therapeutic targets and seek promising and prognostic biomarkers. Previous studies provided evidence supporting the crucial role of COL1A2 in initiation and progression of multiple human cancers, including COAD. However, no studies, to the best of our knowledge, have reported the ceRNA network of COL1A2 and its association with tumor immune infiltration and immune signatures.

In the present study, we first performed pan-cancer expression analysis of COL1A2 in 33 types of human cancers based on TIMER database and integrated data combined TCGA with GTEx. A total of 17 human cancer types with higher COL1A2 levels were selected for prognostic prediction analysis, demonstrating that higher COL1A2 expression indicated unfavorable prognosis in COAD. Previous studies reported the oncogenic role of COLIA1 in colorectal cancer by cell migration, serosal invasion, lymph metastases and hematogenous metastases^12,13^. Consistently, the present data indicates that COL1A2 may serve as a candidate diagnostic biomarker and a promising therapeutic target for COAD.

Accumulating evidence identified the importance of ceRNA network, a new regulatory mechanism in post-transcriptional regulation, in cancer pathogenesis^20^, where ncRNAs participated in regulation of gene expression by talking with each other. miRNAs are single-stranded ncRNAs, generally composed of 21–23 nucleotides^21^ and they generally regulate gene expression by inhibiting mRNA translation or promoting mRNA degradation^22^. We reverse predicted the upstream binding miRNAs of COL1A2 with seven target gene prediction programs in StarBase database, consisting of PITA, RNA22, miRmap, microT, miRanda, PicTar, and TargetScan. The miRNA–mRNA interactions suggest an anticorrelation between a miRNA and its target mRNA, thus R value <  − 0.1 and p value < 0.05 was selected as the threshold in the correlation analysis of prediced miRNAs with COL1A2. As a result, a number of 24 miRNAs were listed as the candidate upstream miRNAs of COL1A2. After performing survival analysis, hsa-miR-552-3p was selected as the most potential upstream tumor suppressive miRNA of COL1A2. Reports on the mechanisms and function identification of hsa-miR-552-3p were lacking. Uniquely, Choi, et al. reported that among 300 miRNAs in an established microRNA library, hsa-miR-552-3p was the most effective in inhibiting cell growth of A549 tumor cells^23^, suggesting that hsa-miR-552-3p may be used as a candidate of cancer inhibitory genes. Our present data conforms to the construction of hsa-miR-552-3p-COL1A2 interaction, which paves the way for further cancer therapeutics.

IncRNAs comprise the main components of ceRNA network, in the way that LncRNAs act as ‘sponges’ for miRNAs, reducing the suppressive effect of miRNAs on target mRNAs^24^. Aberrant IncRNA expression has been linked to developmental disorders and the pathogenesis of many human diseases, including tumors, through a lncRNA-mediated sponge regulatory network of protein-coding driver genes^25^. We used hsa-miR-552-3p to reverse predict its upstream lncRNAs to construct the miRNA-lncRNA network based on the Starbase and LncBase Predicted v.2 databases, and the intersection of the two databases resulted in 16 potential candidate lncRNAs. Based on the ceRNA hypothesis, predicted LncRNAs had positive correlations with COL1A2 expression and played a role of cancer promotion in COAD. By conducting expression analysis, survival analysis, and correlation analysis, LINC00638 was identified as the most potential upstream oncogenic LncRNA of hsa-miR-552-3p. However, rare studies have validated the roles of LINC00638 in driving malignancy and the underlying mechanisms remain to be elucidated. Our present data indicated that LINC00638/hsa-miR-552-3p/COL1A2 axis served as potential regulatory pathways in COAD. The mechanism of this effect requires further study.

The infiltrations of diverse immune cell populations comprise prominent tumor microenvironment components. The cooperation between tumor cells and tumor-infiltrating immune cells shapes therapeutic response and drives tumor development^26^. Our present data demonstrated that increased COL1A2 expression was positively associated with the abundance of CD8+ T cells, CD4+ T cells, macrophages, dendritic cells and neutrophils in COAD. In addition, COL1A2 was also significantly positively associated with biomarkers of these infiltrated immune cells. Our findings suggest that the oncogenic roles of COL1A2 in COAD may be partially attributed to tumor immune infiltration. Furthermore, to gain a deeper understanding of the correlation between COL1A2 and immune infiltration, we performed correlation expression analyses between COL1A2 and various immune signatures encompassing immune cell recruitment and immunomodulation. The positive expression correlation between COL1A2 and chemokines/chemokine receptors partially explains COL1A2-medidated immune cell infiltration. Also worth mentioning is that the expression of COL1A2 was positively correlated with common immunoinhibitors and immunostimulators, indicating that COL1A2 played complex immunological roles in the tumor microenvironment of COAD. However, its main pathways involved in different immunological functions remain to be extensively explored. PD1/PD-L1 and CTLA-4 are widely recognized as crucial immune checkpoints that play a key role in tumor immune escape. In the present work, we confirmed the positive associations between COL1A2 and CD274, CTLA-4, and PDCD1. In the context of tumor progression, COL1A2 overexpression drives immune suppression and contributes to tumor immune escape for COAD, indicating that targeting COL1A2 might be a novel strategy to improve the immunotherapy efficacy of COAD.

In summary, through the comprehensive bioinformatics analyses, we identified high expression COL1A2 in COAD compared to corresponding normal tissues and verified that higher expression of COL1A2 was associated with an unfavorable prognosis for COAD. We have successfully constructed a reverse mRNA prediction model based on LINC00638/hsa-miR-552-3p/COL1A2 ceRNA network as the upstream regulatory mechanism of COL1A2, which advances our understanding of the pathogenesis of this very common tumor and paves the way for further cancer therapeutics. Furthermore, our present data suggested that tumor immune infiltration and tumor immune escape might be involved in COL1A2-medidated cancer development, providing clues for improving the immunotherapy efficacy of COAD by targeting COL1A2. To the best of our knowledge, for the first time, we predicted the ceRNA network of COL1A2 and explored its association with tumor immune infiltration and immune signatures. However, these results should be validated by much more basic experiments and large clinical trials in the future. As we know, ceRNA interactions were multi-target complex network, however, only one link for ceRNA interaction was constructed in the present work, which needs to be validated by much more basic experiments. Also, the cooperation pathways between COL1A2 expression and tumor-infiltrating immune cells require further clarification in the future.

## Methods

### TCGA and GTEx data download, process, and analysis

RNAseq data of COL1A2 in TCGA and GTEx were collected from UCSC XENA (https://xenabrowser.net/datapages/)^27^. Log-transformed expression values after normalization by transcripts per million (TPM) were used for differential expression analysis by Wilcoxon rank sum test. The differential expression between 33 types of cancers (ACC, BLCA, BRCA, CESC, CHOL, COAD, DLBC, ESCA, GBM, HNSC, KICH, KIRC, KIRP, LAML, LGG, LIHC, LUAD, LUSC, MESO, OV, PAAD, PCPG, PRAD, READ, SARC, SKCM, STAD, TGCT, THCA, THYM, UCEC, UCS and UVM) and normal tissues was analyzed for COL1A2 by R package limma^28^. p value < 0.05 was defined as statistical significance.

RNAseq data of mRNA and LncRNA for COAD was collected from TCGA-COAD (https://portal.gdc.cancer.gov/) in terms of level 3 HTSeq-Fregments Per Kilobase per Million (FPKM), which were converted into TPM format for further analyses. Next, level 3 BCGSC miRNA Profiling for COAD was collected from TCGA-COAD in terms of Reads per Million mapped reads (RPM) for miRNA-related analyses. Clinicopathological analyses and molecular mechanism investigations with TCGA-COAD data were performed by R package (V3.6.3).

### TIMER database analysis

TIMER web server (cistrome.shinyapps.io/timer) is a comprehensive resource for systematically investigating molecular characterization of tumor-immune interactions^29^. It provided dynamically displayed figures to conveniently access the tumor immunological features, genomic profile and clinical outcomes. The differential expression of COL1A2 between COAD and normal tissue was explored in “Diff Exp” module. “Gene” module was investigated to estimate the correlations between COL1A2 expression and the abundances of six immune infiltrates (B cells, CD4+ T cells, CD8+ T cells, Neutrophils, Macrophages, and Dendritic cells). “Correlation” module was used to analyze the correlation of COL1A2 expression level with immune checkpoint expression level in COAD.

### GEPIA database analysis

GEPIA is a web-based tool (http://gepia.cancer-pku.cn/) to deliver fast and customizable functionalities for cancer and normal gene expression profiling, clinical outcomes and interactive analyses based on TCGA and GTEx data^30^. The prognostic prediction value of COL1A2 expression level for 17 various cancer types was estimated in the “survival analysis” module, including OS and DFS. Log rank P value < 0.05 was defined as statistical significance. In addition, “correlation analysis” module was investigated to explore the expression correlation of COL1A2 with immune checkpoints (PDCD1, CD274 and CTLA-4) in COAD. Statistical significance was assigned to |R| > 0.1 and p value < 0.05.

### Clinicopathological analysis of COL1A2 in COAD tissues

HPA database provides information of protein-coding gene expression measured by IHC analysis in various cancer tissues and normal tissues (https://www.proteinatlas.org/). We identified immunohistochemically the expression of COL1A2 protein in COAD and colon tissues.

ROC curve was constructed by R package pROC with TCGA-COAD data to estimate the distinguishing efficacy of COL1A2 expression between COAD and normal colon mucosa tissue. In addition, the differential expression of COL1A2 across different pathologic stages (I vs II vs III vs IV) was analyzed with TCGA-COAD data by Kruskal–Wallis test.

### Gene correlation analysis

The top 30 genes negatively and positively correlated with COL1A2 expression in COAD were identified by Spearman correlation coefficient with TCGA-COAD data, and the resulting figures were dynamically displayed in heat maps. In addition, the correlations between COL1A2 and immune signatures including chemokines, chemokine receptors and immunomodular (i.e. immunoinhibitor and immunostimulator) in COAD were analyzed with resulting correlation heat maps. The correlation analysis between COL1A2 and LINC00638 was performed with TCGA-COAD data by R package.

### Functional enrichment analysis of differentially expressed genes

DEGs in COAD grouped by COL1A2 expression level were identified by R package DESeq2 with TCGA-COAD data^31^. The adjusted p value < 0.05 and |log [FC]| > 1.5 were defined as the threshold, and all the DEGs were presented in volcano plots. The functional and pathway differences were explored between the two groups of different COL1A2 expression in COAD using ClusterProfiler R package^32–35^. The values of p.adj < 0.05 and FDR q-value < 0.25 were defined to be statistically significant.

### Candidate microRNA prediction

StarBase (http://starbase.sysu.edu.cn/) is an open-source platform mainly focused on miRNA-target interactions, decoding miRNA-ceRNA, miRNA-ncRNA and protein-RNA interaction networks from large-scale CLIP-Seq data^36^. Upstream binding miRNAs of COL1A2 were predicted with seven target gene prediction programs in StarBase database, consisting of PITA, RNA22, miRmap, microT, miRanda, PicTar, and TargetScan. miRNAs that were predicted by more than two programs were listed as predicted miRNAs^37^. Next, the correlation analysis of predicted miRNAs with COL1A2 was performed with StarBase, and statistical significance was assigned to R value <  − 0.1 and p value < 0.05. Predicted miRNAs meeting the criteria described above were regarded as candidate miRNAs of COL1A2. The prognostic value of candidate miRNAs in COAD were investigated by R package with TCGA-COAD data. Finally, hsa-miR-552-3p was identified.

### Candidate LncRNA prediction

The upstream binding LncRNAs of hsa-miR-552-3p were predicted in the Starbase and LncBase Predicted v.2 databases^38^, and the intersection was taken for further analyses. Guided by the ceRNA hypothesis that IncRNA expression was negatively correlated with targeted miRNA, but positively correlated with targeted mRNA expression, the predicted LncRNA expression, prognostic prediction and correlation analysis with COL1A2 were investigated with TCGA-COAD data. All analyses were considered statistically significant at p < 0.05.

## Data Availability

We acknowledge the TIMER, TCGA, GTEx, GEPIA, HPA, Starbase and LncBase Predicted v.2 databases for free use. All data is available under reasonable request by contacting the corresponding author (Wei Wang). R package (V3.6.3) was utilized.

## Abbreviations

COAD: Colon adenocarcinoma
COL1A2: Collagen type I α 2
COL1A1: Collagen type I α 1
TIMER: Tumor immune estimation resource
TCGA: The Cancer Genome Atlas
GTEx: Genotype-tissue expression
OS: Overall survival
DFS: Disease-free survival
IHC: Immunohistochemical
HPA: Human protein atlas
ROC: Receiver operating characteristic
AUC: Area under the curve
DEG: Differentially expressed gene
FC: Fold change
GO: Gene Ontology
KEGG: Kyoto encyclopedia of genes and genomes
BP: Biological process
CC: Cellular component
MF: Molecular function
ncRNA: Non-coding RNA
GEPIA: Gene expression profiling interactive analysis
TPM: Transcripts per million
FPKM: Fragments per kilobase per million
RPM: Reads per million
